# Self-medication practice and associated factors among adult community members of Jigjiga town, Eastern Ethiopia

**DOI:** 10.1371/journal.pone.0218772

**Published:** 2019-06-28

**Authors:** Mebrahtom Hafte Amaha, Bezatu Mengistie Alemu, Gudina Egata Atomsa

**Affiliations:** 1 Public Health Emergency Management, Ethiopian Somali Regional Health Bureau, Jigjiga, Ethiopia; 2 Departments of Environmental Health Science, Haramaya University, Harar, Ethiopia; 3 School of Public Health, Haramaya University, Harar, Ethiopia; University of Campania, ITALY

## Abstract

**Background:**

Self-medication is the use of any drug or medication to treat an illness or ailment without the supervision of a licensed medical doctor/health care providers. Self-medication practice in Eastern Ethiopia is quite common. However, there is little information with regard to magnitude and associated factors. The objective of this study was to assess the magnitude of self-medication practice and associated factors among adult community members of Jigjiga town, Eastern Ethiopia.

**Methods:**

A community based cross-sectional study was conducted from June 27- July 12, 2017. Multistage sampling method was used and the number of kebeles and Sub-kebeles were selected using simple random sampling technique. Finally, sampled households in the Sub-kebeles were selected using systematic random sampling. Data were collected using face to face interview with 547 adult (≥18 years) participants. It was entered and cleaned using EPI-Data version 3.02 and exported to Statistical Package for Social Science (SPSS) Version 23 for further analysis. Bi-variable and multivariable logistic regression models were carried out to identify factors associated with the self-medication.

**Result:**

The magnitude of self-medication was found to be 37.5% (95% CI: (33.6%–41.7%). Educational status of secondary school [(AOR = 0.46; 95% CI: (0.22–0.98)], high income [(AOR = 3.00; 95% CI: (1.77–5.06)], advised by neighbors, friends or relatives to take drug for their complaint [(AOR = 2.59; 95% CI: (1.62–4.14)], used old prescription /past experience to bought drugs [(AOR = 12.19; 95% CI: (6.65–22.35)], follow advertisements of drugs by television [(AOR = 0.21; 95% CI: (0.05–0.85)], and perception about Hospital drugs (clinics, health centers and hospitals) do not work [(AOR = 2.36; 95% CI: (1.39–3.99)] were significantly associated with self-medication.

**Conclusion:**

High income, advice by neighbors, friends or relatives to take drug for their complaint, old prescription/past experience use to bought drugs, and perception of hospital drugs do not work was positively associated with self-medication. Therefore, health education should be given to the community on the importance of hospital drugs (clinics, health centers and hospitals) to shift their perception.

## Introduction

Self-medication is the use of any drug or medication to treat an illness or ailment without the supervision of a licensed medical doctor [1]. Responsible self-medication can be used to prevent and treat symptoms and ailments that do not need medical consultation or oversight [2]. Butmany people don’t have a clear picture of themselves or their situations and unconsciously fall into a self-medication routine [1]. What’s more, self-medication to a certain extent is not confirmed with regard to interactions, pregnancy, lactation, use in children and the elderly, driving, working conditions, alcohol, or food compared to the medications used with health professional’s prescription[3].

Most people limit their self-medication behaviors to the use of over-the-counter drugs for everyday health complaints such as colds or headaches. However, some people use illicit or illegal substances to self-medicate for serious problems such as severe pain, depression, anxiety disorders or bipolar disorder [1]. It is estimated that a total of 246 million people, or 1 out of 20 people between the ages of 15 and 64 years, used an illicit drug in 2013 [4]. Hence, they can potentially worsen their overall health by developing dependencies on the substances they use[1].

The magnitude of self-medication varies in different studies of different countries. A study conducted in Iran, Jordan, Uganda and Khartoum Sudan shown that the magnitude of self-medication was 53.4%, 39.5%, 75.7% and 81.8% respectively[5–8]. A systematic review on self-medication practice conducted in Ethiopia shown that, the reported prevalence of self-medication in the studies varied from 12.8% (Bahir Dar town residents) to 77.1% (Arsi University health science students), with an overall prevalence of 36.8% [9]. Similarly, a community based studies conducted at Assendabo town, Jimma Zone, Southwestern Ethiopia, Kolladiba Town, North West Ethiopia, and Meket district, Northeast Ethiopia shown that magnitudes of self-medication practice were39.2%, 62.8% and 35.9% respectively [10–12].

Findings of different studies similar to this study revealed that different factor associated with self-medication. A study done in china in four consecutive China National Health Surveys (CNHS) shown that, having ≥5 family member, educational status of high and technical school, and socio-economic status with upper class were found positively associated with self-medication [13]. Likewise, studies done in Uganda and territory of the city of Niš revealed being male sex, long distance to health facility, hospital drugs don’t work, advised by relatives/friends, previous experience/old prescriptions use, and media (television, magazines, and internet) impact on the respondent’s choice regarding self-medication were found risk factors for self-medication [5, 14].

Improper self-medication or medication use may lead to serious adverse drug reactions, overdose and even fatal consequences. Nowadays, there is a global concern about the emergence of drug resistant pathogens which might have been highly augmented by self-medication. Moreover, inappropriate self-medication results in drug dependencies, wastage of resources, and serious health hazards. Self-medication in Eastern Ethiopia is quite common but there is little information regards to the magnitude and associated factors. Therefore, aim of this study was to determine the magnitude and factors associated with self-medication practices among the various segments of the community to deliver appropriate health education, and to halt self-medication related public health problems.

## Materials and methods

### Study design, area and period

A community based quantitative cross-sectional study design was conducted among family members aged 18 years and above of households in Jigjiga town, Eastern Ethiopia from June 27- July 12, 2017. The town is located at eastern part of the country, 628 km far from Addis Ababa the capital city of Ethiopia. Jigjiga town covers with an estimated area of 1.98 square kilometer and it had 20 kebeles constituting 17,001 households [15]. The estimated total population of the Jigjiga town was 257,613. The town had two hospitals (Regional and Referral), 3 health centers and 14 health posts with 1 private hospital, 8 clinics, and 20 drug shops.

### Inclusion and exclusion criteria

#### Inclusion criteria

All family members aged 18 years and above of households in the selected kebeles of Jigjiga town, Eastern Ethiopia.

#### Exclusion criteria

Family members who were seriously ill and unable to respond to the questions during the data collection period.

### Sample size determination and sampling procedure

**Fig 1:**
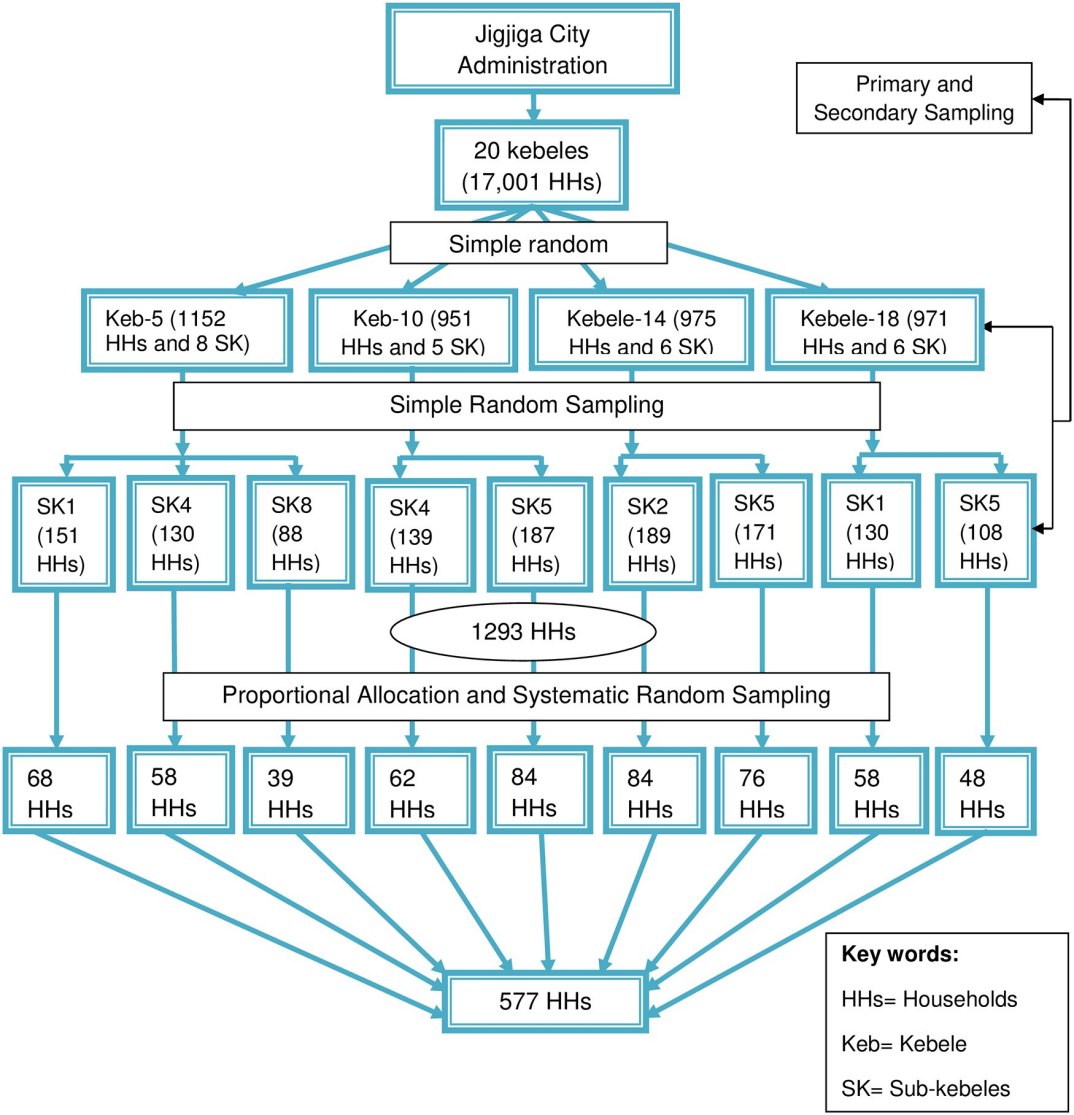
Schematic presentation of Jigjiga town sampling technique.

The sample size of this study was 577 calculated using the parameters: anticipated design effect (1.5) [16], 95% confidence level, 39.2% magnitude of self-medication from the study done in Assendabo town, Jimma Zone, Southwestern Ethiopia [10]. A Multistage sampling method was used. A total of 20 kebeles and 76 sub-kebeles were found in Jigjiga town. Of these, 4 kebeles and 9 sub-kebeles were selected using simple random sampling technique (lottery method). List of all kebeles and sub-kebeles along with their respective household numbers were obtained from Jigjiga City Administration office and considered as primary and secondary sampling unit. Finally, sampled households in the sub-kebeles were selected using systematic random sampling after preliminary survey was done to identify households which contain persons with ailment one month prior to the survey (Fig 1).

### Data collection methods and tools

Face to face interview was conducted with household member’s aged 18 years and above that has faced with perceived ailments in the last one month using structured questionnaire. When more than one individual with perceived ailment is available in one household, one participant was selected by lottery method. Information of the independent variables like socio-demographic characteristics, individual factors, and psychological and health system factors were collected using questionnaire which contains both close-ended and open-ended questions to accommodate all required data. It was translated to Amharic and Af-Somali language by experts for better understanding of the respondents and to ensure consistency. Before data collection, 5% of the questionnaire was pre-tested out of the study area on the same population in a nearby town, Wuchale.

### Operational definitions

In this study self-medication is the selection and use of medicines by individuals to treat self-recognized illnesses or symptoms [17]. It is also defined as the use of traditional medicines and/or modern drugs without consulting qualified health practitioners/without doctor’s prescription. The uses of diet, holy water (“Tsebel”), and other non-pharmacological approaches such as massage, exercise, and psychotherapy was not considered as self-medication [18].

#### Minor illness and long distance to health facilities

In this study minor illness and long distance to health facilities were measured based on the respondent’s perception.

### Data quality control

To attain the data quality control issue training was given for data collectors and supervisors using the local language (Af-Somali). Questionnaire was translated into the local language for data collection and then retranslated back into English version to check consistency. Questionnaire was pre-tested to ensure its consistency and errors were corrected. Short term discussion was held after each data collection day with all data collectors and supervisors to address challenges and to make them clear on how to solve the challenges they faced. Completeness of each questionnaire was checked by the principal investigator and supervisors on daily basis. Double data entry was done by two data clerks and consistency of the entered data was cross checked by comparing the two separately entered data on Epi-Data version 3.02.

### Data processing and analysis

After the data collected and checked for its completeness, it was coded, entered and cleaned using EPI-Data and exported to SPSS. Using the SPSS version 23, first descriptive analysis like frequency, percentage, measures of central tendency and measures of dispersion was carried out. Missing values were analyzed by using multiple imputation technique. Then the information was presented using frequencies tables and figures. Bi-variable logistic regression analysis was done for each variable and variables with their p-value ≤ 0.08 were taken to the multivariable analysis to control all possible confounders. Multi co-linearity test was carried out to see the correlation between independent variables using standard error. However, none of the independent variables were standard error of > 2 to drop one of them. In the multivariable logistic regression analysis the model fitness test was checked using Hosmer and Lemeshow test. Odds ratios along with 95% CI were used to measure association between the dependent and independent variables and level of statistically significant was declared at p-value <0.05.

### Ethical considerations

Ethical approval was obtained from Institutional Health Research Ethics Review Committee (IHREC) of Haramaya University, College of Health and Medical Sciences. A written official letter was taken from IHREC to Ethiopian Somali Regional Health Bureau to deem its legality. After getting permission from Ethiopian Somali Regional Health Bureau a written official letter was also taken to Jigjiga City Administrative offices and each selected kebele and sub-kebele Administrative offices. During data collection each participants were informed about the purpose of the study, why and how they were selected to be involved in the study and what was expected from them. Finally, Written and signed consent were obtained from each participant before data collection and they were informed that they have full right to completely refuse participating in the study, to withdraw from the study at any time and jump questions that they do not want to give response.

## Result

### Socio-demographic characteristics

This study canvassed a total of 577 households with 96.9% response rate. The median ages of the respondents were 31 with inter quartile ranges (IQR) of 25–39 years. More than half of the respondents, 337 (61.6%) were female. Majority 517 (94.5%) of the participants were Muslim followers. More than half, 315 (57.6%) were married, about 241 (44.1%) were attended college and above, and 198 (36.2%) of them were government/NGO employees (Table 1).

**Table 1:** Socio-demographic characteristics of respondents who had illness one month prior to the survey in Jigjiga Town, Eastern Ethiopia, June 27-July 12, 2017 (n = 547).

Characteristic	Category	Self-Medication Practice	
		Yes (%)	No (%)
Sex	Male	83 (40.5)	127 (37.1)
	Female	122 (59.5)	215 (62.9)
Age	18–26 years	72 (35.1)	102 (29.8)
	27–35 years	61 (29.8)	96 (28.1)
	36–44 years	42 (20.5)	96 (28.1)
	45+ years	30 (14.6)	48 (14.0)
Religion	Muslim	195 (95.1)	322 (94.1)
	Orthodox	10 (4.9)	15 (4.4)
	Protestant	-	5 (1.5)
Marital status	Unmarried	65 (31.7)	68 (19.9)
	Married	94 (45.9)	221 (64.6)
	Divorced	24 (11.7)	25 (7.3)
	Widowed	22 (10.7)	28 (8.2)
Educational status	Have no formal education	37 (18.1)	50 (14.6)
	Read and write	20 (9.8)	24 (7.0)
	Primary	22 (10.7)	43 (12.6)
	Secondary	31 (15.1)	79 (23.1)
	College and above	95 (46.3)	146 (42.7)
Occupation	Housewife	38 (18.5)	75 (21.9)
	Daily laborer	20 (9.8)	27 (7.9)
	Government/NGO	76 (37.1)	122 (35.7)
	Merchant	38 (18.5)	68 (19.9)
	Student	33 (16.1)	50 (14.6)
Relationship with the family	Child	44 (21.4)	42 (12.3)
	Husband/wife	134 (65.4)	266 (77.8)
	Relative	27 (13.2)	34 (9.9)
Family size	<2	6 (2.9)	10 (2.9)
	3–4	30 (14.6)	45 (13.2)
	≥5	169 (82.5)	287 (83.9)
Income	Low income	50 (24.4)	132 (38.6)
	Middle income	60 (29.3)	128 (37.4)
	High income	95 (46.3)	82 (24.0)

Monthly income categorized based on PCA (principal component analysis)

### Self-medication practice

**Fig 2:**
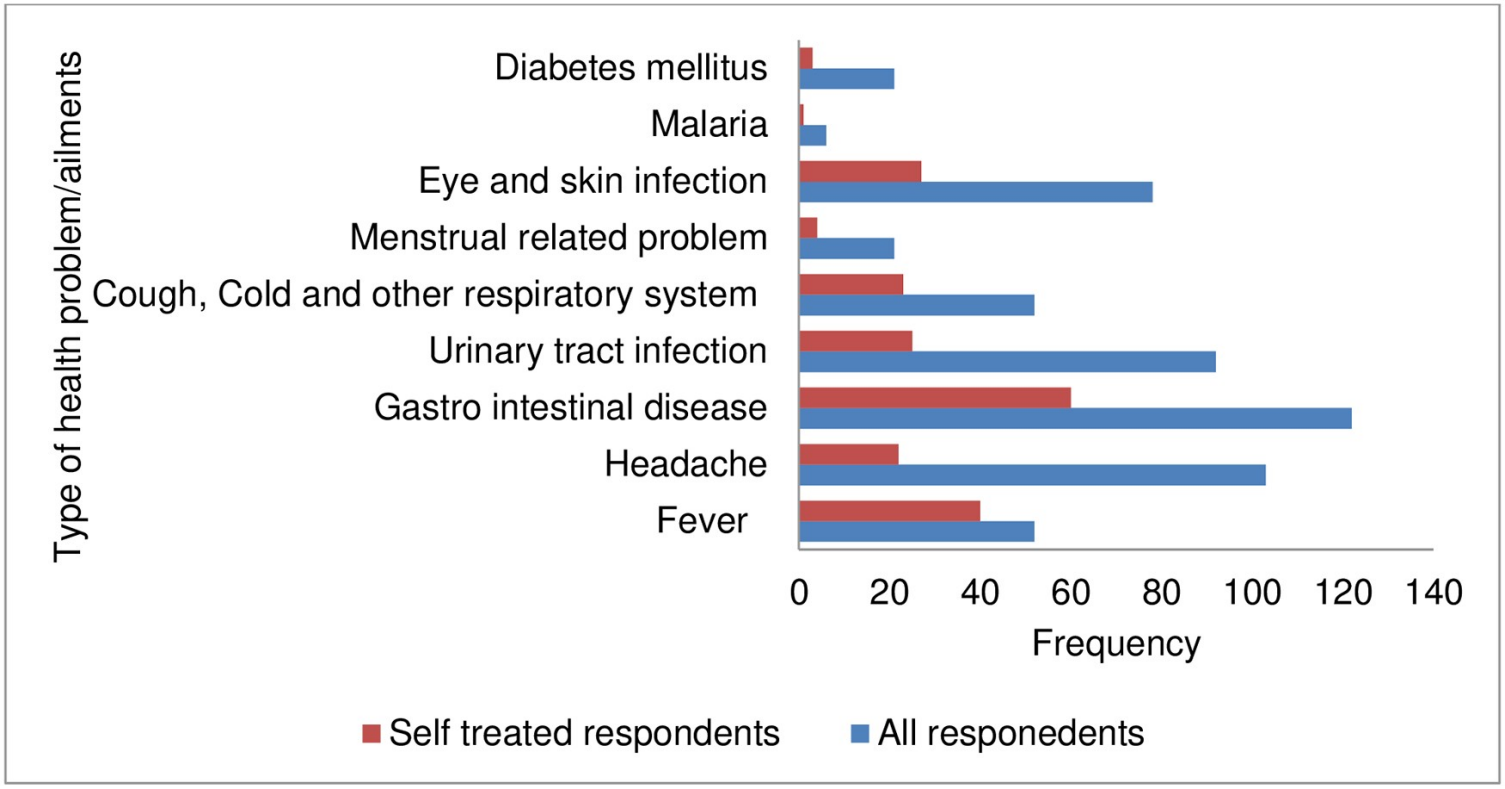
Types of ailments experienced by respondents.

From the ailments reported by respondents, gastrointestinal disease 122 (22.0%), headache 103 (19.0%), urinary tract infection 92 (17.0%), and eye and skin infection 78 (14.3%) were the most reported illness. Whereas gastrointestinal disease 60 (29.3%), Fever 40 (19.5%), and eye and skin infection 27 (31.2%), urinary tract infection 25 (12.2%) were the most reported diseases by respondents practiced self-medication (Fig 2).

**Fig 3:**
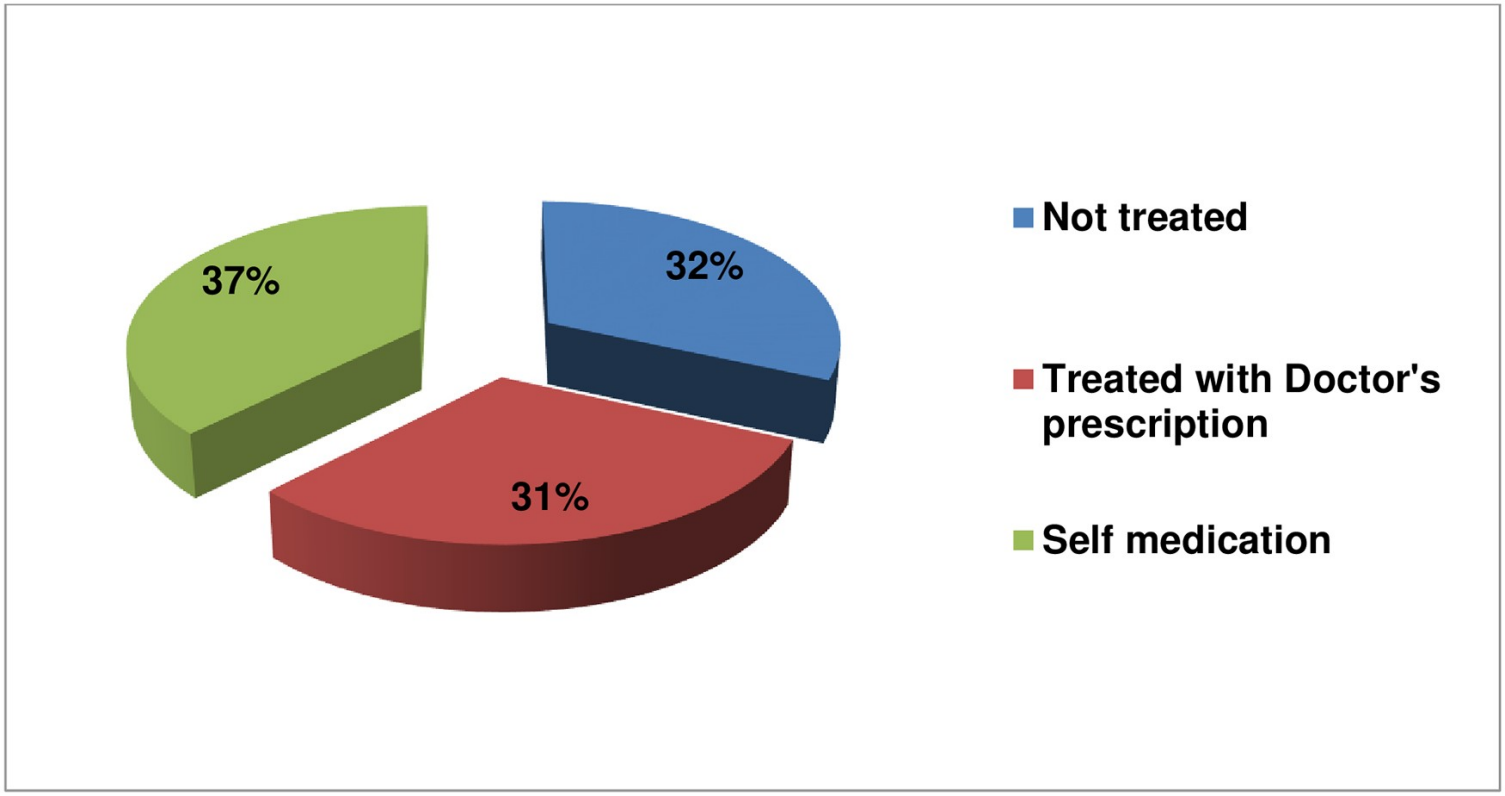
Actions taken by respondents for their perceived ailment.

Regarding to the self-medication practice, from the total 547 respondents 205 (37.5%) has taken medication without doctor’s prescription. Out of the rest 342 (62.5%), 169 (49.4%) of them has not taken drug for their complaint and 173 (50.6%) has treated with doctor’s prescription (Fig 3). From the drugs used for self-medication, antibiotics 84 (41.0%), analgesics 56 (27.3%), Ant-helments 28 (13.7%), and Antacids 25 (12.2%) were frequently used.

Concerning to the place where respondents obtained the drug, more than half 128 (64.4%) of the respondents who has taken drug without doctor’s prescription were obtained from Pharmacy/drug shop followed by fried/relative/neighbors 50 (24.4%).

### Individual factors

More than half of the respondents 323 (59.0%) has taken an advice by neighbors, friends or relatives to take drug for their complaint. However, small numbers of the participants 56 (10.2%) were read label or leaflet or promotional materials of drugs, 46 (8.4%) follow advertisements of drugs by television, 49 (9.0%) use internet to search information about drugs and 40 (7.3%) read magazine related to drugs (Table 2).

**Table 2:** Individual factors of the respondents who had illness one month prior to the survey in Jigjiga Town, Eastern Ethiopia, June 27-July 12, 2017 (n = 547).

Characteristic	Category	Self-Medication Practice	
		Yes (%)	No (%)
Advice by health professionals to take drug for their complaint/symptom	Yes	90 (43.9)	149 (43.6)
	No	115 (56.1)	193 (56.4)
Advice by neighbors, friends or relatives to take drug for their complaint	yes	138 (67.3)	185 (54.1)
	No	67 (32.7)	157 (45.9)
Use old prescription/past experience to bought drugs	yes	76 (37.1)	22 (6.4)
	No	129 (62.9)	320 (93.6)
Read label or leaflet or promotional materials of drugs	yes	15 (7.3)	41 (12.0)
	No	190 (92.7)	301 (88.0)
Follow advertisements of drugs by television	yes	7 (3.4)	39 (11.4)
	No	198 (96.6)	303(88.6)
Use internet to search information about drugs	yes	7 (3.4)	42 (12.3)
	No	198 (96.6)	300 (87.7)
Read Magazine related to drugs	Yes	7 (3.4)	33 (9.6)
	No	198 (96.6)	309 (90.4)

### Psychological and health system factors

More than half of the respondents 367 (67.1%) had minor illness. More than three fourths of the participants 432 (79.0%) reported that taking drug gives quick relief and more than half 324 (59.2%) of them reported that there is overcrowding (long queues) while visiting Doctor/health professionals. Whereas, small number of the respondents reported that 48 (8.8%) health professionals (working in clinics, health centers and hospital) are not well mannered, 46 (8.4%) had chronic disease, 35 (6.4%) health professionals working in health facilities (clinics, health centers and hospitals) are not well trained, and 69 (12.6%) reported fee of drugs in the health facilities (clinics, health centers and hospitals) is expensive. Two hundred sixty three (48.1%) respondents check the expiry dates of drugs during purchasing or before taking and 60 (29.3%) has belief on self-medication/drugs not prescribed by doctor are a good treatment alternative (Table 3).

**Table 3:** Psychological and health system factors of the respondents who had illness one month prior to the survey in Jigjiga Town, Eastern Ethiopia, June 27-July 12, 2017 (n = 547).

Characteristic	category	Self-Medication Practice	
		Yes (%)	No (%)
Minor illness	Yes	131 (63.9)	236 (69.0)
	No	74 (36.1)	106 (31.0)
Health professionals (working on clinics, health centers and hospital) are not well mannered	Yes	21 (10.2)	27 (7.9)
	No	184 (89.8)	315 (92.1)
Taking drug gives you quick relief from your problem/illness	Yes	162 (79.0)	270 (78.9)
	No	43 (21.0)	72 (21.1)
Having chronic disease	Yes	13 (6.3)	33 (9.6)
	No	192 (93.7)	309 (90.4)
Distance to your health facility Long	Yes	58 (28.3)	83 (24.3)
	No	147 (71.7)	259 (75.7)
Health professionals working in health facilities (clinics, health centers and hospitals) are not well trained	Yes	18 (8.8)	17 (5.0)
	No	187 (91.2)	325 (95.0)
Fee of drugs in the health facilities (clinics, health centers and hospitals) is expensive	Yes	27 (13.2)	42 (12.3)
	No	178 (86.8)	300 (87.7)
Hospital drugs (clinics, health centers and hospitals) do not work	Yes	72 (35.1)	59 (17.3)
	No	133 (64.9)	283 (82.7)
Overcrowding (long queues) while visiting Doctor	Yes	134 (65.4)	190 (55.6)
	No	71 (34.6)	152 (44.4)
Checking expiry date of drugs during purchasing or before taking	Yes	99 (48.3)	164 (48.0)
	No	106 (51.7)	178 (52.0)
Belief on self-medication/drugs not prescribed by doctor are a good treatment alternative	Yes	60 (29.3)	-
	No	145 (70.7)	-

### Factors associated with self-medication

Following multivariable analysis the variables were positively and negatively associated with self-medication. Respondents with educational status of secondary school were 54% less likely to practice self-medication compared to those with no formal education [(AOR = 0.46; 95% CI: (0.22–0.98)]. Similarly, respondents who had following advertisements of drug by TV were 79% less likely to practice self-medication compared to those who had not [(AOR = 0.21; 95% CI: (0.05–0.85)].

Participants with high income were 3 times more likely to practice self-medication compared to those with low income [(AOR = 3.00; 95% CI: (1.77–5.06)]. Participants who received an advice by neighbors, friends or relatives to take drug for their complaint were 2.59 times more likely to practice self-medication compare to those who did not received [(AOR = 2.59; 95% CI: (1.62–4.14)]. Participants who had used an old prescription/past experience to brought drug were 12.19 times more likely to practice self-medication compared to those who did not [(AOR = 12.19; 95% CI: (6.65–22.35)]. Likewise, participants who has reported Hospital drugs (clinics, health centers and hospitals) do not work were 2.36 times more likely to practice self-medication compared to those who did not [(AOR = 2.36; 95% CI: (1.39–3.99)] (Table 4).

**Table 4:** Factors associated with self-medication among respondents who had illness one month prior to the survey in Jigjiga Town, Eastern Ethiopia, June 27-July 12, 2017 (n = 547).

Variables	Self-Medication practice		COR (95%CI)	AOR (95%CI)
	Yes (%)	No (%)		
Marital status				
Unmarried	65 (31.71)	68 (19.88)	1.22 (0.63–2.34)	0.74 (0.25–2.16)
Married	94 (45.85)	221 (64.62)	0.54 (0.29–0.99)*	0.56 (0.27–1.16)
Divorced	24 (11.71)	25 (7.31)	1.22 (0.55–2.70)	0.85 (0.32–2.25)
Widowed	22 (10.73)	28 (8.19)	1	1
Educational status				
Have no formal education	37 (18.05)	50 (14.62)	1	1
Read and write	20 (9.76)	24 (7.02)	1.13 (0.54–2.34)	1.55 (0.65–3.71)
Primary	22 (10.73)	43 (12.57)	0.69 (0.36–1.35)	0.59 (0.26–1.36)
Secondary	31 (15.12)	79 (23.10)	0.53 (0.29-.96)*	0.46 (0.22–0.98)*
College and above	95 (46.34)	146 (42.69)	0.88 (0.54–1.45)	0.76 (0.39–1.47)
Relation with the family				
Child	44 (21.46)	42 (12.28)	1	1
Husband/ wife	134 (65.37)	266 (77.78)	0.48 (0.30–0.77)*	0.61 (0.24–1.54)
Relative	27 (13.17)	34 (9.94)	0.76 (0.39–1.47)	0.83 (0.37–1.89)
Income				
Low income	50 (24.39)	132 (38.60)	1	1
Middle income	60 (29.27)	128 (37.42)	0.81 (0.52–1.26)	1.07 (.63–1.82)
High income	95 (46.34)	82 (23.98)	0.33 (0.21–0.51)**	3.00 (1.77–5.06)**
Advice by neighbors, friends or relatives to take drug for their complaint				
Yes	138 (67.32)	185 (54.09)	1.75 (1.22–2.51)*	2.59 (1.62–4.14)**
No	67 (32.68)	157 (45.91)	1	1
Old prescription use/past experience to bought drugs				
Yes	76 (37.07)	22 (6.43)	8.7 (5.11–14.37)**	12.19 (6.65–22.35)**
No	129 (62.93)	320 (93.57)	1	1
Follow advertisements of drugs by television				
Yes	7 (3.41)	39 (11.40)	0.28 (0.12–0.63)*	0.21 (0.05–0.85)*
No	198 (96.59)	303 (88.60)	1	1
Use internet to search information about drugs				
Yes	7 (3.41)	42 (12.28)	0.25 (0.11–0.57)*	1.25 (0.26–6.14)
No	198 (96.59)	300 (87.72)	1	1
Read Magazine related to drugs				
Yes	7 (3.41)	33 (9.65)	0.33 (0.14–0.76)*	0.64 (0.15–2.78)
No	198 (96.59)	309 (90.35)	1	1
Having chronic disease				
Yes	13 (6.34)	33 (9.65)	0.63 (0.33–1.24	0.56 (0.24–10.27)
No	192 (93.66)	309 (90.35)	1	1
Distance to your health facility Long				
Yes	58 (28.29)	83 (24.27)	1.23 (0.83–1.82)	1.01 (0.61–1.68)
No	147 (71.71)	259 (75.73)	1	1
Perception Hospital drugs (clinics, health centers and hospitals) do not work				
Yes	72 (35.12)	59 (17.25)	2.60 (1.74–3.88)**	2.36 (1.39–3.99)*
No	133 (64.88)	283 (82.75)	1	1
Overcrowding (long queues) while visiting Doctor				
Yes	134 (65.37)	190 (55.56)	1.51 (1.05–2.16)*	0.99 (0.61–1.61)
No	71 (34.63)	152 (44.44)	1	1

CI = Confidence Interval, COR = Crude Odds Ratio, AOR = Adjusted Odds Ratio

* = p-value <0.05,

** = p-value<0.001,

## Discussion

The magnitude of self-medication practice was found to be 37.5% (95% CI: 33.6%–41.7%). Pharmacy/drug shops 128 (62.4%) were the main place for respondents to obtain drug for self-medication. Respondents who were secondary school and following advertisements of drugs by television were less likely to practice self-medication. However, participants with high income, advised by neighbors, friends or relatives to take drug for their complaint, used an old prescription/past experience to bought drugs for their complain, and those reported hospital drugs (clinics, health centers and hospitals) do not work were more likely to practice self-medication.

The magnitude of self-medication practice in this study is higher than the finding of the study conducted on three towns of North West Ethiopia which was found to be 27.2%. The reason for this difference could be due to the study conducted on three towns of North West Ethiopia canvassed large number of population to identify individuals with ailment prior to the survey and small number was identified. The same fashion the magnitude of self-medication might become lower than this study [18]. However, it is lower than the study done in Assam, Iraq, and Khartoum, Sudan which was found to be 57.6%, 53.4%, and 81.8% respectively [6, 7, 19]. This gap could be due to the reason that high number of herbal users for self-medication was found in the studies done in Iraq and Khartoum, Sudan [6, 7]. Likewise, the difference with the finding of the study done in Assam could be due to the inclusion of adjacent houses as replacement of locked houses or not willing to answer [19]. Whereas, in this study closed houses were visited three times and if not available, they were considered as no respondents and this might lowered the magnitude of self-medication in this study. On the other hand, it is more or less similar with the finding of the study done in Assendabo town, Jimma zone, southwestern Ethiopia which was found to be 39.2% [10].

In this study the main ailments that led them to practice self-medication were Gastro intestinal disease, fever, eye and skin infection, urinary tracts infection, cough/cold and other respiratory tract infection, and headache. This is consistent with the finding of the studies conducted at Kolladiba Town, North West Ethiopia and three towns of North West Ethiopia [12, 18]. Likewise, it is more or less similar with the findings of the studies conducted in Khartoum, Sudan and Assendabo town, Jimma zone, Southwestern Ethiopia [7, 10]. On the other hand, it is inconsistent with the finding of the study conducted in urban population of Pune, Maharashtra, India that they practice self-medication even for Epilepsy [20]. The reason for this difference might be, in Ethiopia narcotic and psychotropic drugs are prescribed using their own special prescription and audited every three month and individuals with Epilepsy do not get them simply.

In this study, antibiotics, analgesics, anti-helments and antacids were among the major drugs used by respondents to treat self-recognized illness. This finding is more or less similar with the same study done in Sire town, West Ethiopia and Kolladiba Town, North West Ethiopia [12, 21]. However, it is different with the finding of the study done in Dibrugarh town, Assam in which Antihypertensive and Insulin/OHA were among the most relied drugs by the respondents [19]. The reason for this gap might be due the difference in lifestyle and nutritional status.

According to this study finding, respondents with the education level of secondary school were less like to practice self-medication compared to those with no formal education. This finding contrary with the findings of studies conducted in China in four consecutive China National Health Surveys (CNHS) and Jordan [8, 13]. The reason for the gap with the china study finding might be due to the difference in method that the inclusion of all members of the households and many type of self-treatments like drugs and/or other home remedies, or having a massage and/or physiotherapy rather than visiting a physician when experiencing symptoms or complaints during the two-week period preceding the survey [13]. Similarly, the gap with the study conducted in Jordan could be due to the difference in method that was community-based nationwide questionnaire survey conducted on a house-to-house basis [8].

Participants with high income were more likely to practice self-medication compared to the low income. This is similar with the study conducted in China in four consecutive China National Health Surveys (CNHS) [13]. Whereas, it is different with the findings of the study done in Jordan among respondents used antibiotics within the preceding month which reveals respondents with middle incomes were more likely to practice self-medication than low income. This difference might be due to the reason mentioned above that the study conducted in Jordan was community-based nationwide questionnaire survey which might cover large number of participants [8].

The finding of this study showed that participants who got advice from neighbor/friends/relatives, used old prescription/past experience and those with perception of hospital drugs do not work were more likely to practice self-medication. This finding is similar with the study done in Northern Uganda among adult antimicrobial users [5]. A study conducted in Meket district, Northeast Ethiopia similarly shown that respondents who had peer/family pressure and past experience of self-medication were more likely to practice self-medication compared to those who had not [11]. Likewise, this study revealed that respondents who were following advertisements of drug by TV were less likely to practice self-medication. This could be due to the short comedies and health educations about the side effects of improper use of drugs given by Ethiopian Somali TV. Though association not occurred, in this study the relationship between internet use to search information about drugs and self-medication was assessed. This result contradicts with the findings of the studies conducted in Italy, which indicates internet and social media are widely used by the participants to search health-related information [22, 23]. The season for this difference could be due the limited internet accesses in the study area.

This study tried to assess the magnitude and factors associated with self-medication and this will help as baseline information for other researchers to study the consequences self-medication practice among Jigjiga Communities. However, it might be influenced by limitations: like recall bias because the data was collected with one month ailments recall and respondents with chronic disease might expect curative drug.

## Conclusion

More than one thirds of the respondents practiced self-medication. Educational status of secondary school, high income, advised by neighbors, friends or relatives to take drug for their complaint, used old prescription/past experience to buy drug, following advertisements of drug by TV and perception of hospital drugs (clinics, health centers and hospitals) do not work were significantly associated with self-medication practice. Therefore, health education should be given to the community on the importance of hospital drugs (clinics, health centers and hospitals) to shift their perception. Likewise, further studies need to be carried out to study the consequences of self-medication practice and to include it to the surveillance system.
